# Biocompatible and colloidally stabilized mPEG-PE/calcium phosphate hybrid nanoparticles loaded with siRNAs targeting tumors

**DOI:** 10.18632/oncotarget.6428

**Published:** 2015-11-28

**Authors:** Pei Gao, Xiangyu Zhang, Hongzhi Wang, Qinghong Zhang, He Li, Yaogang Li, Yourong Duan

**Affiliations:** ^1^ State Key Laboratory for Modification of Chemical Fibres and Polymer Materials, College of Materials Science and Engineering, Donghua University, Shanghai 201620, China; ^2^ State Key Laboratory of Oncogenes and Related Genes, Shanghai Cancer Institute, Renji Hospital School of Medicine, Shanghai Jiao Tong University, Shanghai 200032, China; ^3^ Traditional Chinese Medicine Department, Renji Hospital, School of Medicine, Shanghai Jiao Tong University, Shanghai 200127, China

**Keywords:** calcium phosphate, nanoparticle, siRNA delivery, biocompatible

## Abstract

Calcium phosphate nanoparticles are safe and effective delivery vehicles for small interfering RNA (siRNA), as a result of their excellent biocompatibility. In this work, mPEG-PE (polyethylene glycol-L-α-phosphatidylethanolamine) was synthesized and used to prepare nanoparticles composed of mPEG-PE and calcium phosphate for siRNA delivery. Calcium phosphate and mPEG-PE formed the stable hybrid nanoparticles through self-assembly resulting from electrostatic interaction in water. The average size of the hybrid nanoparticles was approximately 53.2 nm with a negative charge of approximately −16.7 mV, which was confirmed by dynamic light scattering (DLS) measurements. The nanoparticles exhibited excellent stability in serum and could protect siRNA from ribonuclease (RNase) degradation. The cellular internalization of siRNA-loaded nanoparticles was evaluated in SMMC-7721 cells using a laser scanning confocal microscope (CLSM) and flow cytometry. The hybrid nanoparticles could efficiently deliver siRNA to cells compared with free siRNA. Moreover, the *in vivo* distribution of Cy5-siRNA-loaded hybrid nanoparticles was observed after being injected into tumor-bearing nude mice. The nanoparticles concentrated in the tumor regions through an enhanced permeability and retention (EPR) effect based on the fluorescence intensities of tissue distribution. A safety evaluation of the nanoparticles was performed both *in vitro* and *in vivo* demonstrating that the hybrid nanoparticle delivery system had almost no toxicity. These results indicated that the mPEG-PE/CaP hybrid nanoparticles could be a stable, safe and promising siRNA nanocarrier for anticancer therapy.

## INTRODUCTION

Due to the ability of small interfering RNA (siRNA) to effectively silence specific genes, siRNA has quickly emerged as one of the most promising drugs for the treatment of various human diseases, such as viral infections, genetic diseases and cancer [1–4]. This treatment method is based on living cells introducing exogenous genes, which encode therapeutic proteins that can correct and eradicate diseases at the sources [5]. However, naked siRNA is rarely delivered to target cells resulting in silenced genes because of ribonuclease (RNase) degradation, inefficient cellular uptake and rapid clearance from systemic circulation upon intravenous injection [6–8]. Therefore, the development a safe and efficient delivery system for siRNA remains the main challenge for the clinical success of RNA interference (RNAi) therapy. A large number of non-viral vectors have been developed to pack siRNA molecules into nanoparticles, including cationic lipids, [9, 10] polymers [11, 12] and inorganic nanoparticles [13–15].

Calcium phosphate (CaP) is the main mineral found in human bone and teeth and is considered as a highly biocompatible inorganic biomaterial [16–18]. CaP precipitates are able to effectively encapsulate negatively charged nucleic acids and are a suitable candidate for use as an siRNA carriers [19, 20]. Moreover, amorphous CaP is sparingly soluble in water and can be rapidly synthesized by mixing aqueous solutions of calcium and phosphate ions [21–23]. CaP rapidly dissipates in acidic pH conditions, and CaP endocytosed by cells is degraded in the endosomes and releases siRNA into the cytoplasm [24, 25]. However, uncoated CaP colloids are very unstable and tend to aggregate to form large particles. The cellular uptake of large CaP particles is limited and the large agglomerates cannot be used for therapeutic applications *in vivo* [26–28]. Thus, the preparation of stable CaP nanoparticles is critical for the delivery of siRNA to target tissues *in vivo*.

Many researchers have attempted to prepare stable CaP colloids for systemic siRNA delivery. Recently, Li et al. developed CaP nanoparticles with lipid coatings for siRNA delivery where the outer lipid stabilied the CaP core [29]. Xie et al. reported the development of PEGylated carboxymethyl chitosan and CaP anionic nanoparticles to deliver siRNA [30]. In addition, Lee et al. prepared dopa-hyaluronic acid conjugate CaP nanoparticles for target-specific delivery of siRNA [31]. Therefore, PEGylated CaP can used to generate safe and stable hybrid nanoparticles for efficient siRNA transfection *in vivo*.

Phospholipids are a major component of cell membranes and are widely used to prepare drug carriers. Anionic phospholipids are negatively charged and can complex with CaP through electrostatic interactions [32–34]. In this work, anionic L-α-phosphatidylethanolamine was utilized as a hydrophobic moiety and mPEG was used to hydrophilic moiety to formed anionic mPEG-PE block copolymers. mPEG-PE block copolymers can self-assemble into anionic micelles in water, which are able to form hybrid nanoparticles with cationic CaP and control CaP growth (denoted as NP_mPEG-PE/CaP_). CaP can condense siRNA into the hybrid nanoparticles and mPEG is able to prolong the circulation time of nanoparticles (denoted as NP/siRNA). NP_mPEG-PE/CaP_ can deliver siRNA into cancer cells and target tissues through EPR effects. Moreover, the safety of NP_mPEG-PE/CaP_ has been evaluated *in vivo*. Due to the non-cytotoxic nature of both CaP and mPEG-PE, there is great value in developing NP_mPEG-PE/CaP_ as an siRNA delivery system.

## RESULTS

### Synthesis and characterization of mPEG-PE

**Figure 1:**
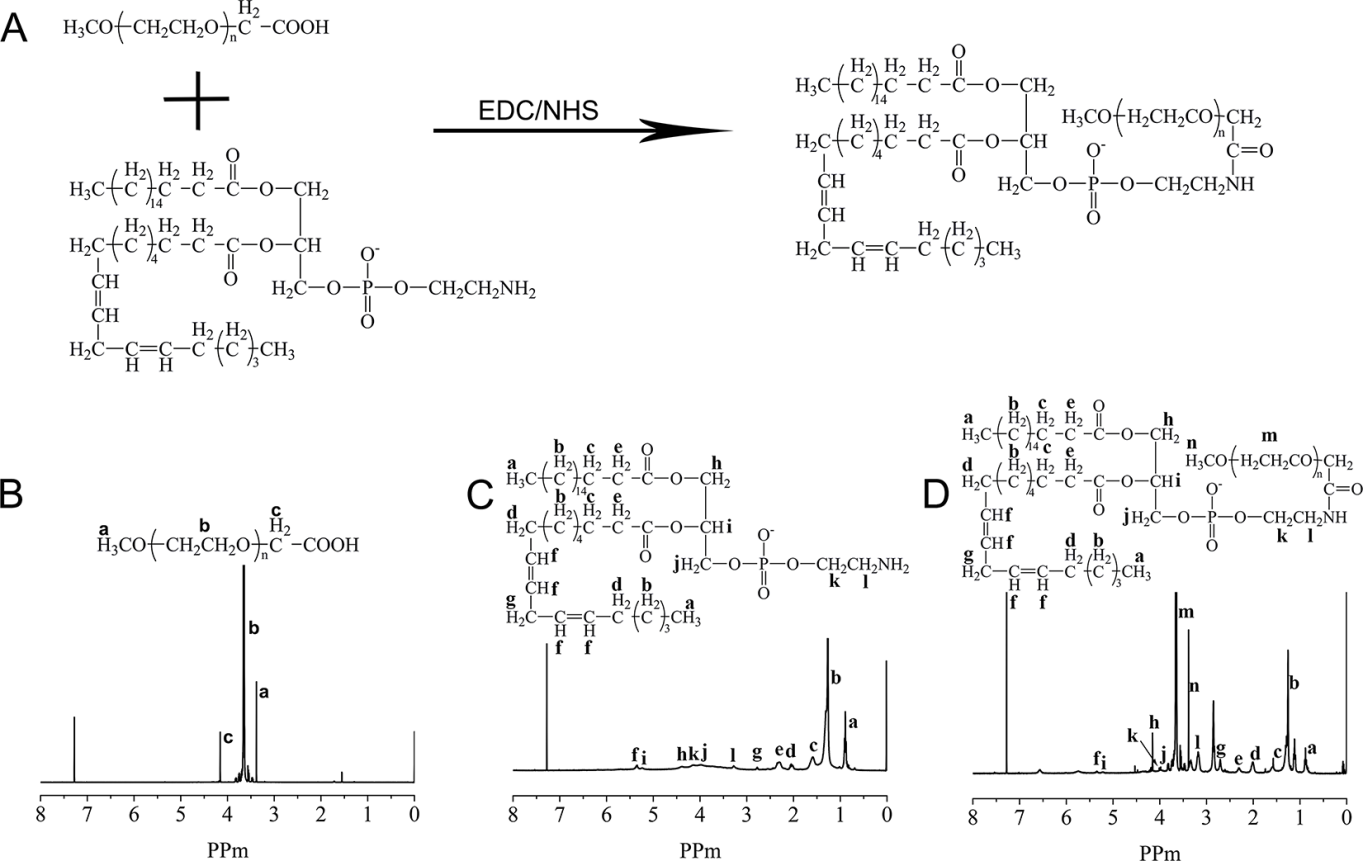
Synthetic route for mPEG-PE (**A**). ^1^H NMR spectra of mPEG-COOH (**B**), PE (**C**) and mPEG-PE (**D**).

The synthetic route to prepare mPEG-PE is illustrated in Figure 1A. The amine groups of PE was reacted with the carboxyl groups of mPEG-COOH to form mPEG-PE [35, 36]. The reaction was performed in TCM and the EDC/NHS was used as a coupling agent to reduce reaction times. The residual PE and EDC/NHS was removed with anhydrous diethyl ether. The structure of mPEG-PE was confirmed by FT-IR spectra and ^1^H NMR spectra. The ^1^H NMR spectra of mPEG-PE is shown in Figure 1D. The proton peak of PE (−CH_3_ at δ 0.9, −CH_2_− at δ 1.3, −CH = CH− at δ 5.34) (Figure 1C) were observed in the spectra of mPEG-PE. The existence of methoxy group (CH_3_O− at δ 3.4) and the PEG segment (−CH_2_CH_2_O− at δ 3.7) (Figure 1B) peaks from mPEG-COOH demonstrated successful conjugation.

**Figure 2:**
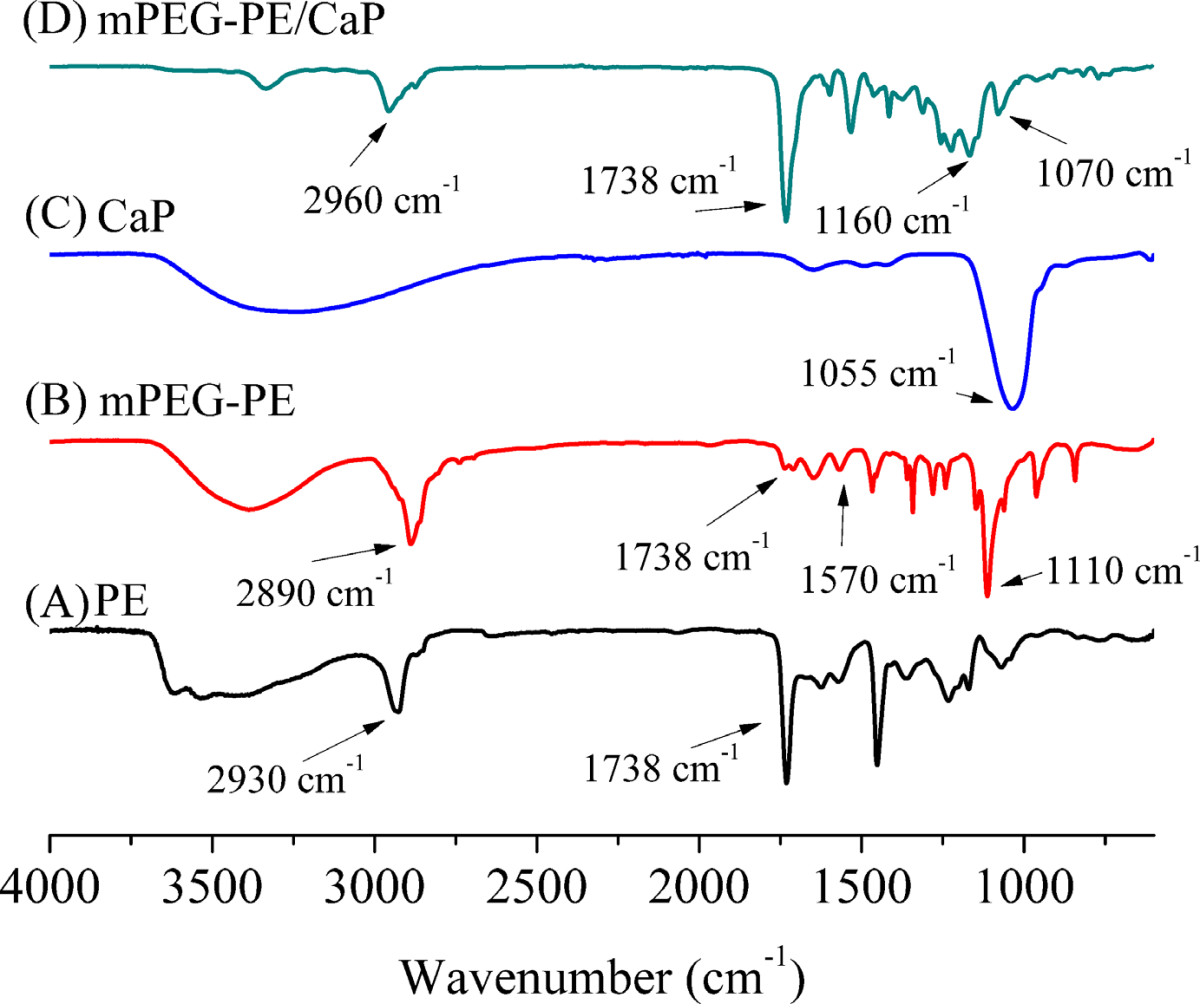
Infrared spectroscopy of PE (**A**), mPEG-PE (**B**), CaP (**C**) and mPEG-PE/CaP (**D**).

FT-IR analysis demonstrated that the C = O groups appeared at 1738 cm^−1^ in the IR of PE and mPEG-PE (Figure 2). The characteristic peaks of CO-NH and C-O-C were present at approximately 1570 cm^−1^ and 1110 cm^−1^, respectively. These results further indicated the successful synthesis of mPEG-PE.

### Preparation and characterization of NP_mPEG-PE/CaP_

**Figure 3:**
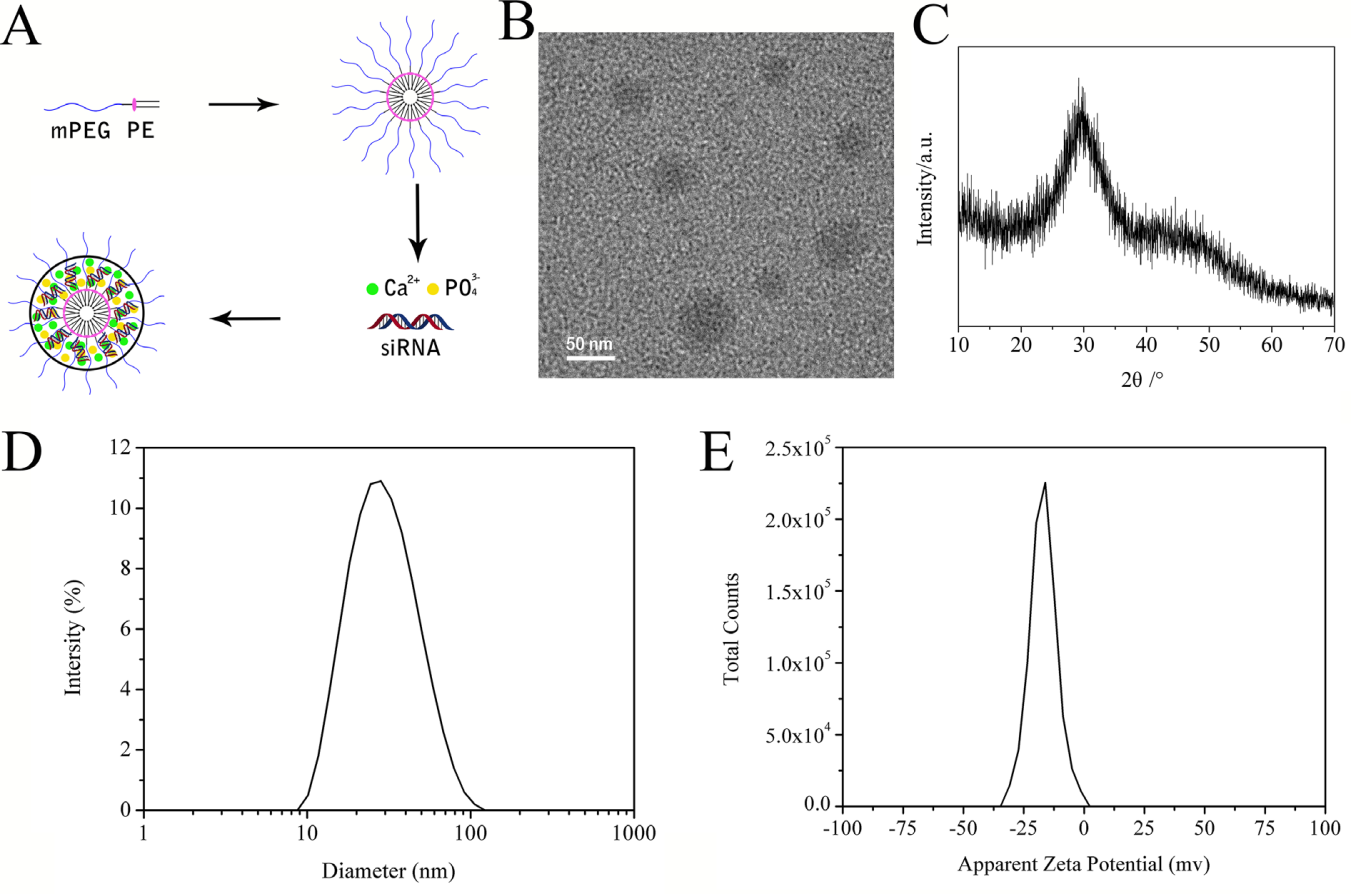
Schematic illustration of mPEG-PE/CaP/siRNA hybrid nanoparticles (**A**). TEM image of NP/siRNA nanoparticles (**B**). XRD of NP/siRNA nanoparticles (**C**). Diameter distribution of mPEG-PE/siRNA nanoparticles (**D**). Zeta potential of NP /siRNA (**E**).

The mPEG-PE micelles were generated using a thin film hydration method with the amphiphilic block copolymer. The anionic micelles were formed through the self-assembly of a thin film of the mPEG-PE in an aqueous buffer solution (Figure 3A). The hydrophobic PE segments formed the dense inner core of micelles, while the hydrophilic mPEG segments formed the outer shell. This preparation method was simple and repeatable.

To package siRNA into the anionic mPEG-PE micelles, calcium ions were used as electrostatic bridges between siRNA and the mPEG-PE micelles. The hybrid nanoparticles were prepared by mixing a solution of phosphate ions with a solution containing mPEG-PE, siRNA and calcium ions (Figure 3A). The calcium ions, phosphate ions and siRNA self-assembled into the micelles to form the PEG-coated CaP nanoparticles.

The FT-IR spectra of NPmPEG-PE/CaP are shown in Figure 2. Peaks at 1055 cm^−1^ are attributed to −PO_4_ in amorphous CaP that was prepared in the absence of mPEG-PE (Figure 2C). Figure 2D shows the FTIR the peaks of C-O-C, C = O and −CH_2_ at approximately 1160 cm^−1^, 1738 cm^−1^ and 2960 cm^−1^, respectively. Moreover, peaks at 1070 cm^−1^ were observed in the FTIR spectra of NP_mPEG-PE/CaP_, which originated from −PO_4_. These results demonstrated that the mPEG-PE and CaP hybrid nanoparticles were successfully prepared.

The morphology of the nanoparticles was observed by TEM, which showed that the nanoparticles were spherical and had no obvious aggregation (Figure 3B). The particle sizes of NP/siRNA were characterized by DLS. As shown in Figure 3D, the average size was approximately 53.2 ± 1.8 nm and the polydispersity index was 0.124, both of which were beneficial for the passive tumor targeting of drug delivery through the EPR effect. The nanoparticles in aqueous solution were negatively charged with a zeta potential of −16.7 ± 0.8 mV (Figure 3E).

The XRD patterns of NP/siRNA confirmed the absence of distinct of crystalline CaP peaks. As shown in Figure 3C, a peak characteristic peak of an amorphous phase was observed at 2θ = 30°, indicating that the nanoparticles consisted of the amorphous calcium phosphate [37].

### Colloidal stability study of NP/siRNA

**Figure 4:**
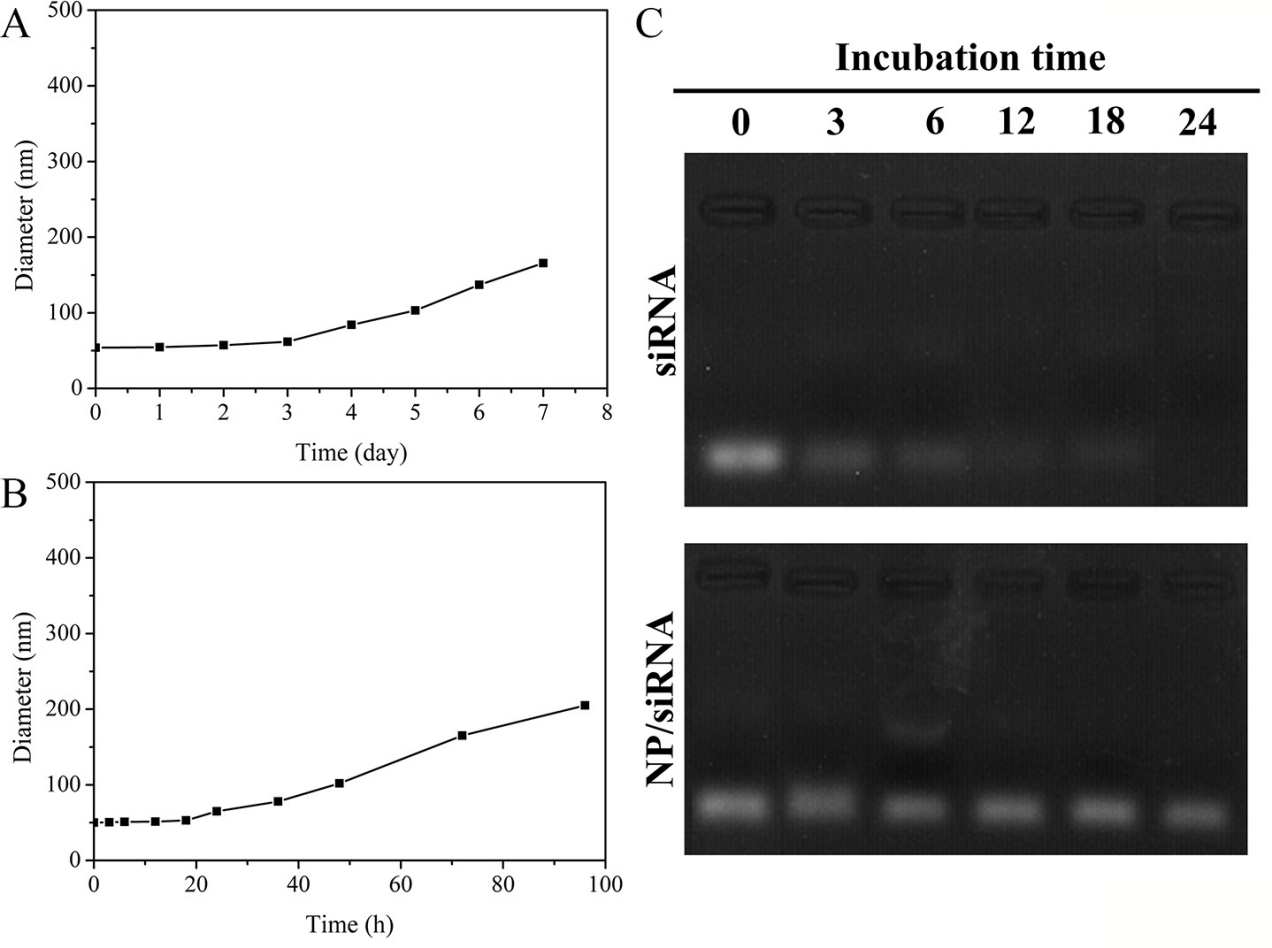
Changes in particle sizes of NP/siRNA following incubation at 4°C (**A**). and 37°C in serum (50%) (**B**). Values are shown as the mean ± SD (*n* = 3). Gel retardation assay of siRNA and NP /siRNA in serum at 37°C for different periods of time (**C**).

Particle aggregation negatively affects the longer circulation times of intravenously administered particles. The stability of the generated nanoparticles was evaluated by measuring the mean diameter of the particles. As shown in Figure 4A, the size of the nanoparticles ranged from 53 nm to 168 nm after being stored for seven days at 4°C. Moreover, the colloidal stability of nanoparticles in FBS was investigated. The size of the hybrid nanoparticles was monitored in a solution containing 50% FBS at 37°C. The size hardly any increased in FBS, as demonstrated after incubation for 24 h (Figure 4B). However, the size of the nanoparticles increased to 205 nm after 96 h. These results suggested that the surface PEG corona of the nanoparticles effectively prevented particle aggregation and enhanced their colloidal stability.

To evaluate the serum stability of the hybrid nanoparticles loaded with siRNA when circulating in blood, the degradation of siRNA and NP/siRNA were studied in 50% FBS at 37°C with a gel retardation assay. It was found that NP_mPEG-PE/CaP_ could protect siRNA from RNase degradation in serum for 24 h (Figure 4C). However, the naked siRNA was quickly degraded after 6 h of incubation. These results clearly demonstrate that NP_mPEG-PE/CaP_ is suitable for the systemic delivery of siRNA.

Effective encapsulation of siRNA in the nanoparticles was confirmed by a centrifugal assay, which indicated that about 80% of siRNA were loaded in the hybrid nanoparticles. Due to the CaP condensing nucleic acids, the hybrid nanoparticles had very high encapsulation efficiencies.

### Cellular uptake of NP/Cy3-siRNA

**Figure 5:**
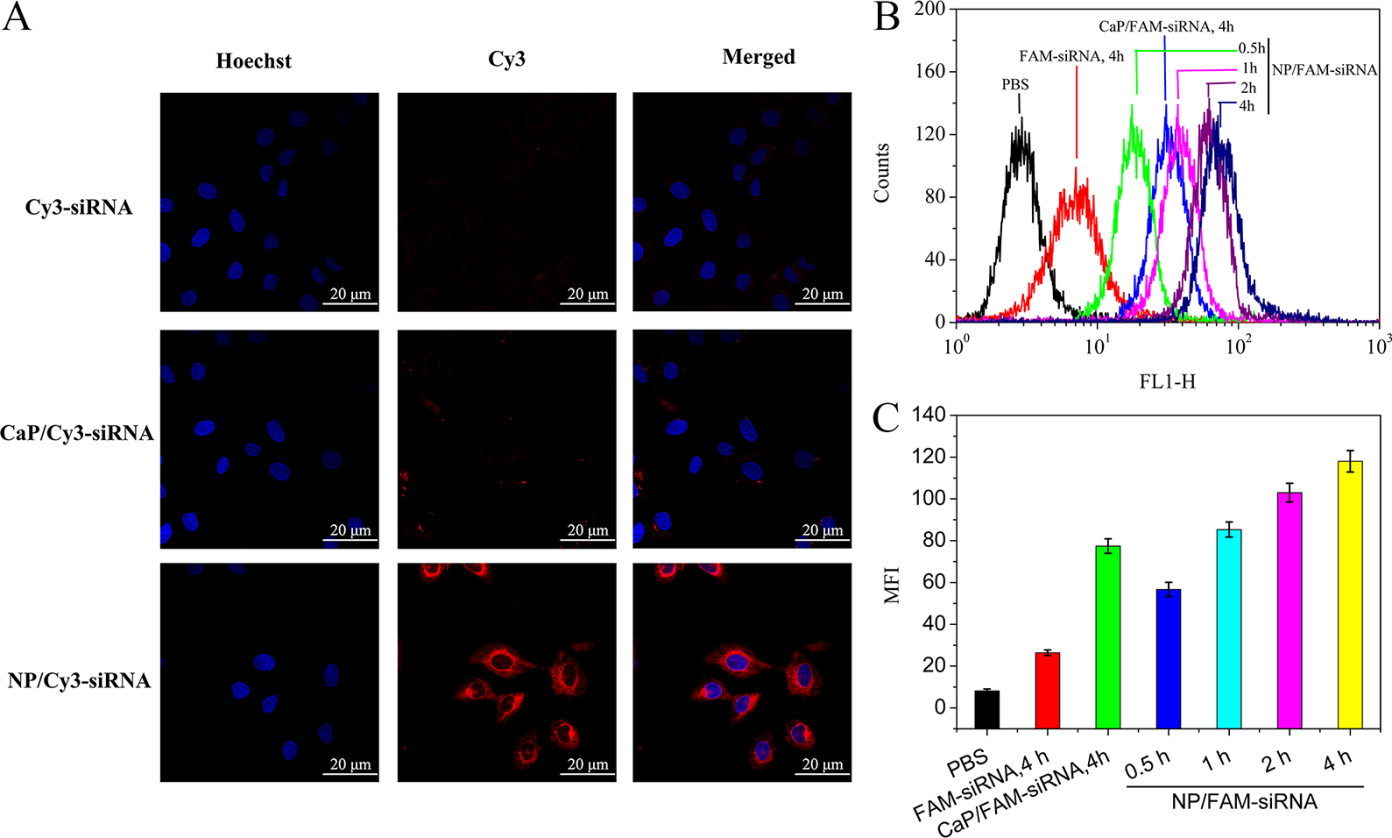
Confocal laser scanning microscopic (CLSM) images of SMMC-7721 cells incubated with Cy3-siRNA, CaP/Cy3-siRNA and NP/Cy3-siRNA for 4 h (**A**). Flow cytometry analyses of the cellular uptake of CaP/Cy3-siRNA, NP/FAM-siRNA (**B**) and the corresponding percentages of FAM-siRNA (**C**).

The internalization and intracellular distribution of the hybrid nanoparticles in SMMC-7721 cells were observed with confocal microscopy. Cy3-labelled siRNA was used as a fluorescent probe, and the cell nuclei were stained with Hocehst 33342. As shown in Figure 5A, CaP precipitated and formed large aggregates, which reduced their transfection efficiencies. Cy3-siRNA- and Cy3-siRNA-loaded CaP and NP_mPEG-PE/CaP_ were observed by CLSM after incubation with cells for 4 h. Based on flow cytometry data, the intracellular fluorescence intensity of FAM-siRNA uptake by SMMC-7721 cells for different periods of time were observed in the following order: NP_mPEG-PE/CaP_ (4 h) > NP_mP_EG-PE/CaP (2 h) > NP_mPEG-PE/CaP_ (1 h) > CaP (4 h) > NP_mPEG-PE/CaP_ (0.5 h) > free FAM-siRNA (4 h) (Figure 5B and 5C). Compared to the free FAM-siRNA, NP_mPEG-PE/CaP_ could strongly enhanced the cellular uptake of siRNA.

### *In vivo* biodistribution study

**Figure 6:**
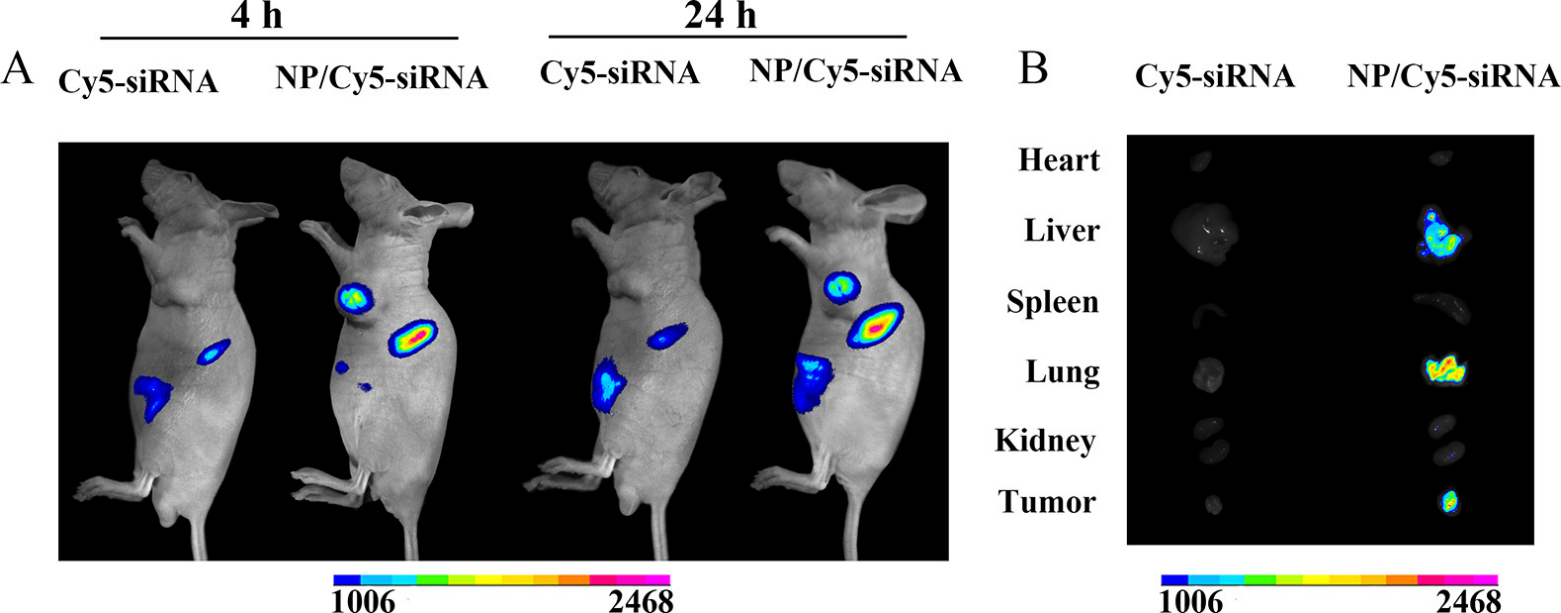
*In vivo* distribution of Cy5-labelled siRNA in nude mice bearing xenografted SMMC-7721 tumor at 4 h and 24 h after intravenous injection of nanoparticles (**A**). Fluorescence images of excised tumors and organs at 24 h (**B**).

To observe the biodistribution of mPEG-PE/siRNA/CaP nanoparticles in SMMC-7721 tumor-bearing mice, Cy5-siRNA loaded in NPs and free Cy5-siRNA were injected into the mice at doses of 1.2 mg/kg. The *in situ* fluorescence distributions were monitored by fluorescence imaging at 4 h and 24 h. As shown in Figure 6A, free Cy5-siRNA was quickly eliminated in mice 4 h after injection. Compared to free Cy5-siRNA, the Cy5-siRNA encapsulated NPs accumulated in tumors within 4 h and were distinctly observed up to 24 h post-injection at the tumor site. Subsequently, the tumor and major organs were collected and imaged after the mice were sacrificed at 24 h post-injection. As shown in Figure 6B, no fluorescence signals were observed in the mice treated with free Cy5-siRNA during the experimental period. The tumor tissues showed significant fluorescence signals. In addition, the fluorescence signals were stronger in the liver and the lung.

### Safety evaluation

**Figure 7:**
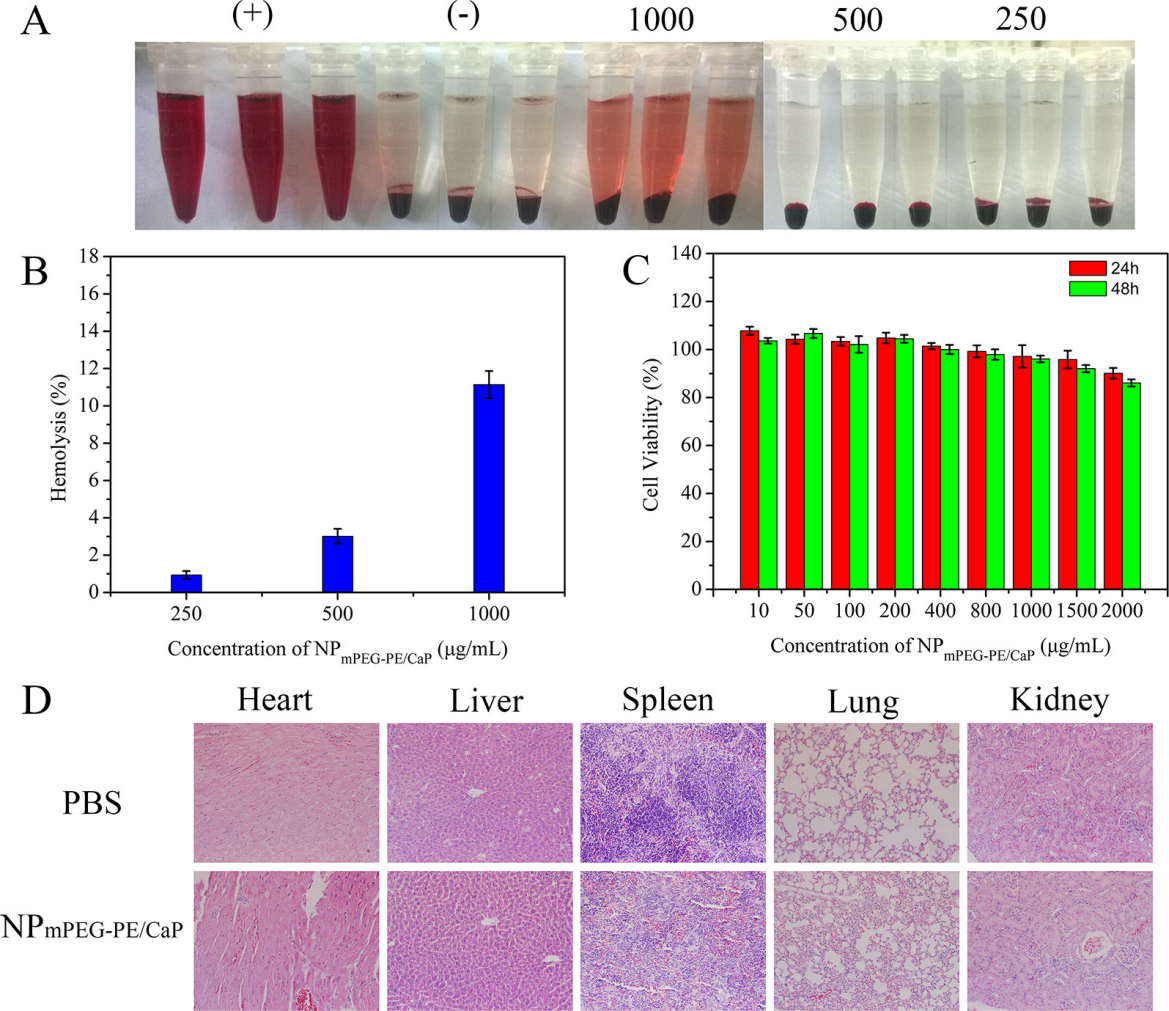
Digital photos illustrating the haemolysis effects of RBCs after 1 h coincubation with NP_mPEG-_PE/CaP at different concentrations (**A**) (*n* = 3). Haemolysis percentages of RBCs incubated with NP_mPEG-PE/CaP_ (**B**). Cell viability of NP_mPEG-PE/CaP_ with different concentrations in SMMC-7721 cells (**C**). Histopathology of H&E-stained major organs from mice after intravenous injections of PBS and nanoparticles (**D**).

The haematological biocompatibility of NP_mPEG-PE/CaP_ was evaluated using a haemolytic experiment *in vitro*. The dark red colour of the positive control suggested the rupture of red blood cells but the negative control was transparent and the red blood cells were deposited at the bottom of the tube (Figure 7A). The colors of the NP_mPEG-PE/CaP_ samples at different concentrations were between the negative control and positive control and exhibited much lower haemolytic effects.

The cytotoxicity of NP_mPEG-PE/CaP_ was evaluated in SMMC-7721 cells via an MTT assay. As shown in Figure 7C, no significant cytotoxicity was observed for the cells treated with NPs when the final concentration of NP_mPEG-PE/CaP_ was 2000 μg/mL or less.

The systemic toxicity of NPs was investigated after intravenous administration to ICR mice once every other day for a total of three times. The mice exhibited good activity and death was not observed during treatment. Blood samples and major organs were collected and harvested for haematology and histological analysis. As shown in Table 1, compared to mice injected with the PBS, there was little elevation of AST, ALT, BUN and Cr in the blood serum. The intravenous injection of NPs elicited no remarkable toxicity in the liver and kidney. In addition, the levels of white blood cells (WBC), RBCs, haemoglobin (HGB), platelets (PLT), neutrophils (NEU), lymphocytes (LYM), and monocyte (MON) were measured (Table 2). The above parameters for the nanoparticles showed no dramatic changes compared with those of the PBS group. As presented in Figure 7D, the H&E-stained sections of the main organs were observed. There were no significant histological differences between the main organs of the nanoparticle-treated and PBS-treated mice.

**Table 1:** Serum levels of AST, ALT, BUN and Cr at 24 h after intravenous injections of PBS and nanoparticles

Sample	AST (U/L)	ALT (U/L)	ALP (U/L)	Cr (μmol/L)
PBS	110 ± 2.34	45.5 ± 2.15	277 ± 13.17	11 ± 0.65
NPs	98 ± 3.13	36.5 ± 3.45	288 ± 18.45	10 ± 0.74

Values shown are as the mean ± SD (*n* = 3).

**Table 2:** Haematological parameters after treatment with PBS and nanoparticles

Sample	WBC (10^9^/L)	RBC (10^12^/L)	HGB (g/L)	PLT (10^9^/L)	NEU (10^9^/L)	LYM (10^9^/L)	MON (10^9^/L)
PBS	4.8 ± 0.32	6.99 ± 0.12	152 ± 0.41	803 ± 43.20	0.45 ± 0.02	4.56 ± 0.57	0.02 ± 0.01
NPs	5.4 ± 0.45	6.97 ± 0.22	152 ± 0.34	741 ± 35.56	0.65 ± 0.07	4.63 ± 0.63	0.06 ± 0.03

Values shown are the mean ± SD (*n* = 3).

## DISCUSSION

To prepare stable colloidal CaP nanoparticles, mPEG-PE was synthesized to generate anionic micelles. The calcium ions and phosphate ions can self-assembled to condense siRNA to the mPEG-PE micelles and formed stabilized nanoparticles. Simple CaP precipitation was difficult to control, and bulky agglomerates formed naturally [38]. The outer mPEG of the nanoparticles successfully inhibited the CaP precipitates from increasing in size and could facilitated longer circulation times for the intravenously administered particles. Moreover, the PEG chains inhibited CaP forming crystalline long-range order structures [39]. In contrast to crystalline CaP, the amorphous nanoparticles were bioactive and exhibited enhanced biodegradability [40–42]. In addition, the nanoparticles with a zeta potential of −16.7 ± 0.8 mV avoided forming lager aggregates in the presence of negatively charged serum proteins in blood [43].

**Scheme 1:**
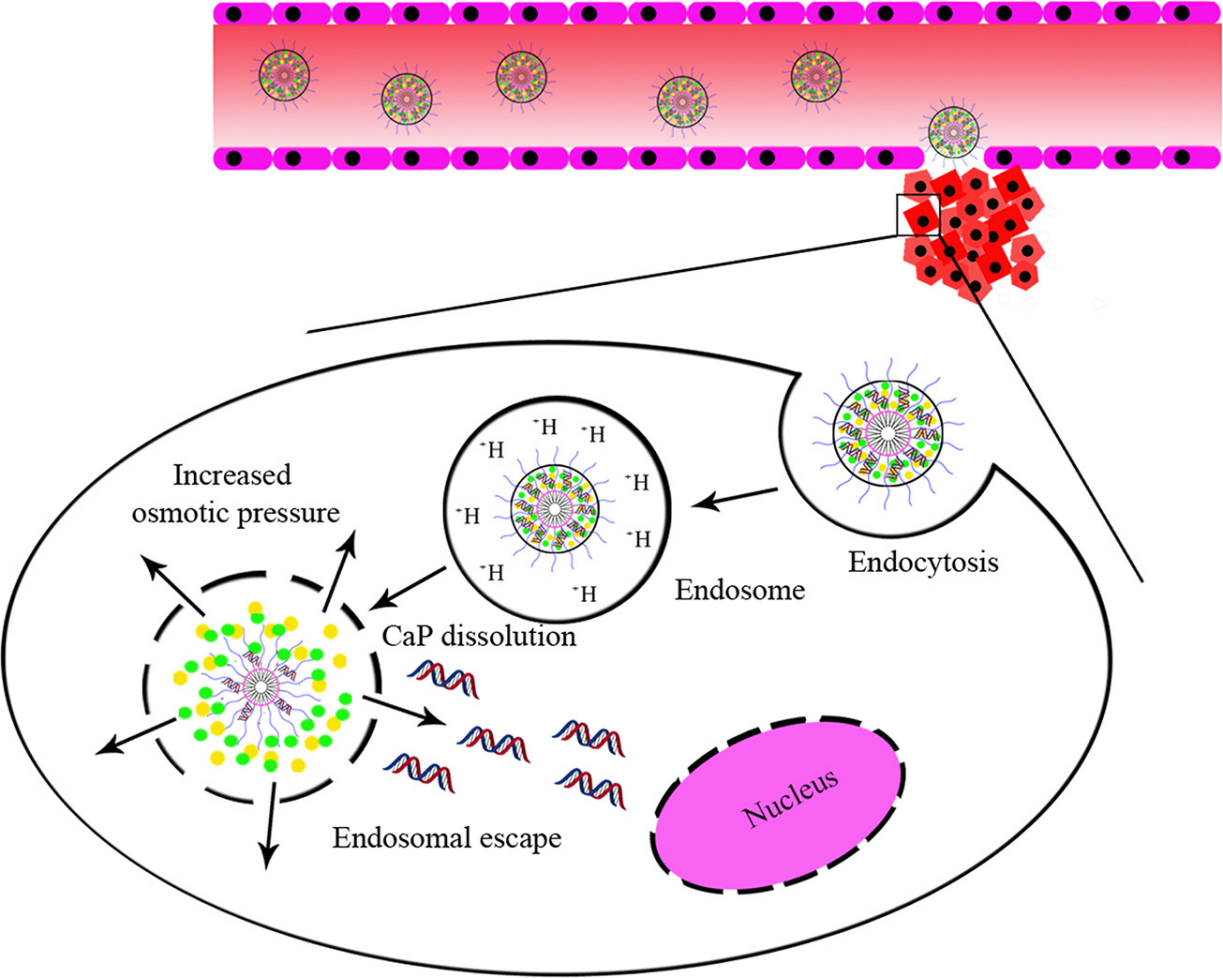
NP_mPEG-PE/CaP_ for the systemic delivery of siRNA and cellular uptake.

Compared with free siRNA and CaP-loaded siRNA, the hybrid nanoparticle-loaded siRNA could efficiently deliver siRNA to cells *via* endocytosis. To investigate the gene silencing efficiency of siRNA, siRNA must be released to the cytoplasm after the cellular uptake of the hybrid nanoparticles with siRNA. As shown in Scheme 1, the hybrid nanoparticles in the acidic endosome may increase internal osmotic pressure due to the dissolution of CaP, leading to swelling and disruption of the endosomal membrane. Cy5-siRNA encapsulated NPs could be protected from RNase degradation, obviously enhancing Cy5-siRNA accumulation in tumor tissue through the EPR effect and quickly releasing Cy5-siRNA in intracellular acidic conditions (Scheme 1). The higher accumulation of nanoparticles in the lung may have been a result of the aggregates lodging in fenestrated capillaries. Some particles may have adsorbed to different types or amounts of serum proteins and became entrapped in the capillary bed of the lung [44–47]. The fluorescence signal was weaker in the liver because most of the nanoparticles were not captured by the mononuclear phagocyte system (MPS) and the reticuloendothelial system (RES) in the liver.

CaP is considered the most biocompatible inorganic biomaterial and is a suitable candidate drug carriers as it is the main mineral found in human bone and teeth [48, 49]. Moreover, natural cephalin is a major component of cell membranes and is considered an excellent biocompatibile biomaterial. The biocompatibility of hybrid nanopariticles was studied by testing haemolytic activity, cytotoxicity and systemic toxicity. The results of this study demonstrated that the haemolysis percentage of NP_mPEG-PE/CaP_ at the highest concentration of 1000 μg/mL was still low (12%) due to the mPEG on the surface of NP_mPEG-PE/CaP_. The nanoparticles possessed a negligible cytotoxicity even at hybrid nanoparticles concentrations of 2000 μg/mL. In addition, the intravenous injection of nanoparticles induced no obvious toxicity under the experimental conditions. Overall, mPEG-PE and CaP hybrid nanoparticles are suitable candidates for drug carriers due to their excellent biocompatibility.

In conclusion, an amphiphilic mPEG-PE copolymer was synthesized by conjugating PE with mPEG-COOH. We have developed a hybrid nanocarrier system consisting of CaP and an mPEG-PE copolymer for safe and efficient siRNA delivery. It was demonstrated that mPEG-PE/CaP nanoparticles showed stable properties, good biocompatibility and effective cell uptake. Moreover, the vehicles can delivered siRNA to liver cancer cells *in vivo* and did not activate an innate immune response. Consequently, the mPEG-PE/CaP hybrid nanoparticles are promising siRNA loading systems thatcan be used for cancer treatment.

## MATERIALS AND METHODS

### Materials

L-α-phosphatidylethanolamine (Soy) was purchased from Avanti Polar Lipids, Inc. (Alabaster, AL). mPEG-COOH was purchased from Shanghai Yare Biotech, Inc. n-Hydroxysuccinimide (NHS) and 1-Ethyl-3-(3-dimethylaminopropyl)carbodiimide hydrochloride (EDC·HCl) were obtained from GL Biochem (Shanghai) Ltd. Hepes, sodium hydroxide (NaOH), tris(hydroxylmethyl)aminomethane (Tris), 3- (4, 5-dimethylthiazol-2-yl)-2, 5-diphenyltetrazolium bromide (MTT) and hydrochloric acid (HCl) were purchased from Sigma-Aldrich. Calcium chloride (CaCl_2_), trisodium phosphate (Na_3_PO_4_), sodium chloride (NaCl) and trichloromethane (TCM) were obtained from Sinopharm Chemical Reagent Co., Ltd. Foetal bovine serum (FBS), trypsin, penicillin, streptomycin, and high-glucose Delbecco's modified Eagle medium (DMEM) were obtained from Biosera.

The human liver cancer cell line SMMC-7721 was obtained from the Institute of Biochemistry and Cell Biology at the Chinese Academy of Sciences (Shanghai, China). SMMC-7721 cells were cultured in DMEM containing 10% FBS and 1% penicillin/streptomycin at 37°C in a humidified atmosphere containing 5% CO_2_.

Nude Balb/c mice (6 weeks old) were purchased from the Shanghai Silaike Laboratory Animal Ltd (Shanghai, China). All care and handling of animals were performed according to a research protocol approved by the Animal Care and Use Committee of the Shanghai Cancer Institute.

### Synthesis and characterization of mPEG-PE

mPEG-COOH (1 g) and PE (0.4 g) were dissolved in 4 mL of TCM. After the mixture was stirred for 1 h at 25°C, and EDC·HCl (192 mg) and NHS (115 mg) were added to the solution. The solution was allowed to react for 24 h at room temperature and was subsequently poured into anhydrous diethyl ether. The precipitate was washed twice with anhydrous diethyl ether, dried under vacuum and stored at 4°C. After drying under vacuum, mPEG-PE was obtained as a yellow powder.

The chemical structure of mPEG-PE was confirmed by nuclear magnetic resonance (NMR) spectroscopy and Fourier transform infrared (FT-IR) spectra. ^1^H NMR spectra were measured using a Bruker Avance 400 (400 MHz) spectrometer in deuterated chloroform (CDCl_3_). FT-IR spectra were recorded on a Fourier transform infrared spectrometer (Nicolet NEXCU 670).

### Preparation and characterization of NP/siRNA

mPEG-PE (40 mg) was dissolved in 4 mL TCM and dried under vacuum. After evaporating the TCM, the residual membrane was dispersed in 4 mL of Tris-HCl (10 mM Tris-HCl pH 7.4) to form mPEG-PE micelles. The micelles were stored at 4°C.

siRNA (150 μL of 20 μM) was added to 100 μL of a 100 mM CaCl_2_ solution, and was subsequently mixed with 250 μL of an mPEG-PE (1 mg/mL) solution. HBS (500 μL; 50 mM Hepes, 280 mM NaCl, 1.5 mM Na_3_PO_4_, pH = 7.4) was quickly added to the mPEG-PE/Ca^2+^/siRNA solution and was allowed to react for 30 min. To remove the excess Ca^2+^, the reaction mixture was centrifuged at 1000 g and 4°C for 1 h using Amicon^®^ Ultra-4 centrifugal filter devices (MWCO: 10 kDa). The sample solution was used in further experiments.

The particle size, polydispersity index (PDI) and zeta potential of the prepared nanoparticles were determined using dynamic light scattering (DLS) (Malvern Zetasizer nano ZS, Malvern) measurements. To observe the morphology of the nanoparticles, the nanoparticle solution was dropped onto a 300-mesh carboncoated copper grid and the excess solution was removed using a filter paper. The grid was allowed to dry at room temperature and was observed using transmission electron microscopy (TEM) (H-800, Hitachi, Japan). X-ray diffraction (XRD) measurements were carried out on a Rigaku D/Max-2550PC X-ray diffractometer using Cu Kα radiation.

### Stability of NP/siRNA and encapsulation efficiency of siRNA in nanoparticles

The nanoparticles with siRNA encapsulation were incubated at 4°C and then further incubated with 10% FBS (v/v) at 37°C. At predetermined time points, the samples were collected and analysed using a gel retardation assay. The siRNA and NP/siRNA were loaded into a 1% agarose gel containing 0.5 μg/mL ethidium bromide. Electrophoresis was performed at 100 mV for 15 min and the resulting gels were visualized using a GelDoc XR imaging system (Bio-Rad Laboratories Ltd.). The mean diameter of the nanoparticles was monitored at predetermined intervals by a Malvern Zetasizer Nano ZS.

To determine the encapsulation efficiency of siRNA, the nanoparticles solution was centrifuged at 15000 g and 4°C for 30 min. The nanoparticles were precipitated out and the fluorescence of the supernatant liquid was measured at 260 nm using a spectrophotometer (NanoDrop 2000, Thermo Scientific). The encapsulation efficiency of siRNA was calculated as follows:

Encapsulation efficiency = 100 − (Abs_260_ after centrifugation)/(Abs_260_ before centrifugation) × 100% [50].

### Cellular uptake of NP_mPEG-PE/CaP_

SMMC-7721 cells were cultured with 1 mL DMEM containing 10% FBS on 20-mm glass-bottom dishes (NEST) at 5 × 10^4^ cells/dish. After 24 h, the medium was exchanged with fresh medium, and NP/Cy3-siRNA, CaP/Cy3-siRNA and Cy3-siRNA were added to the dish (100 nM Cy3-siRNA) for different amounts of time. The SMMC-7721 cells were washed 3 times with PBS and were stained with Hoechst33342 for 5 min. The intracellular distribution of nanoparticles was visualized with a FV-1200 Olympus confocal microscope.

SMMC-7721 cells (1 × 10^5^) were seeded onto 6-well plates with DMEM containing 10% FBS. After 24 h, the medium was replaced with fresh medium containing NP/FAM-siRNA nanoparticles (100 nM FAM-siRNA). At different time points, the cells were washed with PBS and detached with trypsin. The cellular uptake of FAM-siRNA was monitored using flow cytometry (FACSCalibur, BD Bioscience).

### *In vivo* distribution of NP_mPEG-PE/CaP/Cy5-siRNA_

To construct animal tumor models, SMMC-7721 cells (1 × 10^6^) were subcutaneously injected into the flank region of female nude mice (approximately 20 g). When the tumors grew to approximately 500 mm^3^ in size, mice were intravenously injected with free Cy5-siRNA and NP/Cy5-siRNA at a Cy5-siRNA dose of 1.2 mg/kg. Fluorescence imags were acquired at the predetermined intervals, using a fluorescence imaging system (LB 983, Berthold Technologies Gmbh & Co.KG).

### Cytotoxicity and haemolysis assay

Red blood cells (RBCs) were obtained from rat blood by centrifugation at 2000 r/min for 10 min at 4°C and were washed for three times with a PBS solution. Then, the RBCs were diluted to 20% (v/v) of their volume with PBS. RBC solution (100 μL) was added into 400 μL of the NP_mPEG-PE/CaP_ solution at different concentrations, and the resulting solutions were incubated at 37°C for 1 h. Deionized water was used as a positive control and pure PBS was used as a negative control. Then, the samples were centrifuged at 4000 r/min for 5 min, and the supernatant liquid was measured using a UV-Vis spectrophotometer at λ = 541 nm. The haemolysis percentages of NP_mPEG-PE/CaP_ were calculated as follows:

$$\text{Hemolysis}\%=({\text{Abs}}_{\left(\text{sample}\right)}-{\text{Abs}}_{\left(-\right)})/({\text{Abs}}_{\left(+\right)}-{\text{Abs}}_{\left(-\right)})$$

SMMC-7721 cells were seeded on 96-well plates (5000 cells/well) with 100 μL DMEM containing 10% FBS. After a 24 h incubation, NP_mPEG-PE/CaP_ was added with fresh medium. After 24 h or 48 h, 100 μL of DMEM containing 0.5 mg/mL MTT was added, and the resulting solution was incubated for an additional 4 h. The medium was replaced with 150 μL DMSO. The absorbance of each well was measured at 490 nm using a microplate reader (Multiskan FC, Thermo).

### Systemic toxicity assay

The hybrid nanoparticles solutions were intravenously injected into the mice once every two days for a total of three times. The blood and major organs were collected at 24 h after the last injection. Blood routine tests were performed and the levels of alkaline phosphatase (ALP), aspirate aminotransferase (AST), alanine aminotransferase (ALT) and creatinine (Cr) were measured in serum samples. Major organs including the heart, liver, spleen, lung and kidney were fixed and processed thereafter for haematoxylin and eosin (H & E) staining.
